# Characteristics of Small-Molecule Migration of Silicone Rubber Insulator in Electrical Power Systems

**DOI:** 10.3390/polym14132519

**Published:** 2022-06-21

**Authors:** Xiaobo Meng, Gongmao Peng, Kang Niu, Xiaogang Wang, Hongwei Mei, Liming Wang

**Affiliations:** 1 School of Mechanical and Electrical Engineering, Guangzhou University, Guangzhou 510006, China; mengxb@gzhu.edu.cn; 2 Tsinghua Shenzhen International Graduate School, Tsinghua University, Shenzhen 518055, China; gmpeng@sz.tsinghua.edu.cn (G.P.); niuk@sz.tsinghua.edu.cn (K.N.)

**Keywords:** pollutants, kaolin, kieselguhr, small-molecule migration, high-temperature vulcanizing, hydroxyl angle

## Abstract

The migration of low-molecular-weight components of polysiloxane (small molecules) to the surface of high-temperature-vulcanizing silicone rubber (HTV-SR) ensures its hydrophobicity and tends to coat the surface of pollutants, which would otherwise lower hydrophobicity. The transferability of hydrophobicity will ensure the insulator maintains its higher hydrophobicity after being coated with surface pollutants, thus providing the insulator with higher pollution flashover voltage. This migration process takes a certain time, and in this paper, the time characteristics of hydrophobicity transfer from HTV-SR coated with ten different inert materials were investigated. Ten different inert materials have different migration times and static contact angles, possibly due to the influence of pollution material characteristics on the characteristics of small-molecule migration. Thermogravimetric analysis (TG), Fourier transform infrared spectroscopy (FTIR), and gas chromatography–mass spectrometry (GC–MS) were used to analyze the migration of small molecules to the polluted surface. The evidence of small molecules migrating to the surface of the polluted material over time was found. Furthermore, the influence of pollution material characteristics on small-molecule migration was analyzed via SEM, specific surface area, and porosity. On that basis, the hydrophobicity migration characteristics of mixtures of kaolin and kieselguhr were also studied and compared to determine how best to simulate the behavior of natural pollution using artificial pollutants and their mixtures.

## 1. Introduction

Excellent hydrophobicity and unique migration effects are found in high-temperature-vulcanizing silicone rubber (HTV-SR). Hence, the composite insulators made of HTV-SR are widely used in transmission lines [1,2,3]. A theoretical hypothesis exists indicating that hydrophobicity originates in the migration of the low-molecular-weight (LMW) polysiloxane to the surface of the silicone rubber [4,5,6]. HTV-SR has a wide range of molecular weights, and LMW components can move through the bulk material relatively easily. LMW components form a film on the surface, and consequently, the surface acquires hydrophobicity. Even when the surface loses hydrophobicity due to severe weather conditions, the hydrophobicity state recovers with time due to further LMW migration [7].

The range of molecular weights of the silicone rubber inevitably varies depending on the batch formula, and the actual degree of hydrophobicity will vary with external factors such as the degree of pollution and environmental factors such as severe weather [8,9,10,11,12]. Different pollutants coating silicone rubber change its hydrophobicity and lead to changes in insulation performance. The hydrophobicity and insulation performance of silicone rubber coated with a different pollutant ensures that the maximum pollution flashover voltage is sustained in the electrical power system [13,14]. The higher pollution flashover voltage can protect the insulator from pollution flashover in various environments and not lead to power failure. However, whether small molecules can migrate to the surface of the pollution is unclear [12]. Hence, the evidence of small molecules migrating to the surface of the pollution layer over time should also be found. Additionally, the influence of pollution material characteristics on small-molecule migration is also unclear [15]. Therefore, the influence of pollution material characteristics on small-molecule migration needs further study to expand the application of silicone rubber in different polluted environments.

In the midst of a considerable number of pollutants, kaolin and kieselguhr were very typically and often used. These two materials have been recommended as pollutants for artificial pollution tests on ceramic and glass insulators in the IEC standard [16]. However, the influence of pollution material characteristics (kaolin and kieselguhr) on the characteristics of small-molecule migration was not analyzed in depth [17,18,19]. Differences in the rate at which the LMW components of silicone rubber migrate to the surface of the pollution should be studied to determine whether the mixtures of kaolin and kieselguhr can be used as artificial pollutants instead of natural pollution in the pollution flashover voltage test, as the pollution flashover voltage tests for composite insulators are not yet included in international standards and due to “their applicability to composite insulators not having been proven” [20].

In this paper, the time characteristics of the hydrophobicity transfer from HTV-SR coated with ten different inert materials were investigated. Ten different inert materials have different migration times and static contact angles, possibly due to the influence of pollution material characteristics on small-molecule migration. Thermogravimetric analysis (TG), Fourier transform infrared spectroscopy (FTIR), and gas chromatography–mass spectrometry (GC–MS) were used to analyze the migration of small molecules to the polluted surface. The evidence of small molecules migrating to the surface of the pollution over time was found. Furthermore, the influence of pollution material characteristics on the characteristics of small-molecule migration was analyzed using SEM, specific surface area, and porosity. On that basis, the hydrophobicity migration characteristics of mixtures of kaolin and kieselguhr were also studied and compared to determine how best to simulate the behavior of natural pollution using artificial pollutants and their mixtures.

## 2. Experimental Arrangement and Measurement Mothed

**Figure 1:**
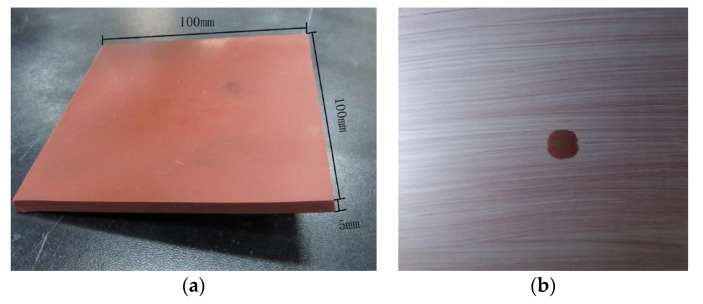
Test piece of HTV silicone rubber: (**a**) clean surface; (**b**) surface coated with pollutant.

A typical high-temperature-vulcanizing silicone rubber (HTV-SR) sample is shown in Figure 1a. The definition of the high-temperature-vulcanizing silicone rubber (HTV-SR) could be referred to in the literature [12]. The pollutants were applied to the surface using the solid layer method [16]. A contaminant solution consisting of pollutants and water was used to coat the sample surfaces, as shown in Figure 1b. The non-soluble deposit density (NSDD) in each case was 10 μg/mm^2^. In addition to kaolin, kieselguhr, and their mixtures, the other pollutants used were powdered coal, charcoal, zinc oxide, aluminum, cement, silica, and two types of natural pollution. These latter comprised pollutants collected from the upper surfaces of overhead transmission line insulators within 1000 m of the sea and a steel plant. These pollutants were then dissolved in deionized water to remove the salt contamination by the filter paper.

**Figure 2:**
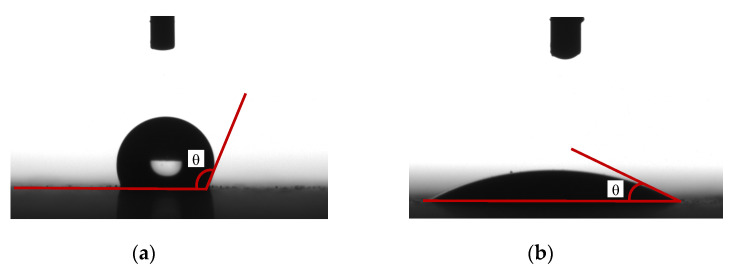
Water droplets on silicone rubber surfaces: (**a**) hydrophobic and (**b**) hydrophilic.

There are currently two methods available for determining the hydrophobicity of HTV silicone rubber [21]. The first is to determine hydrophobicity classification (HC) via the spray method, and the second is the contact angle (CA) method. The spray method is generally used to assess the hydrophobicity of insulators on site but is easily affected by subjective factors. Another disadvantage of the HC determination is that it does not allow a quantitatively comparative analysis, so the CA method was used in the present study. Ten droplets of distilled water, each of about 10 μL, were allowed to fall randomly onto the HTV-SR sample. The static contact angle was obtained from side-view photographs of the droplets by an optical contact angle measuring instrument (OCA20, DataPhysics, Germany) [9,10], as shown in Figure 2. The contact angles of the droplets’ left and right sides were determined using a built-in program developed by DataPhysics. The contact angle was the angle between the HTV surface and the tangent line of the droplet drawn at the triple junction between air, the HTV surface, and the water droplet.

**Figure 3:**
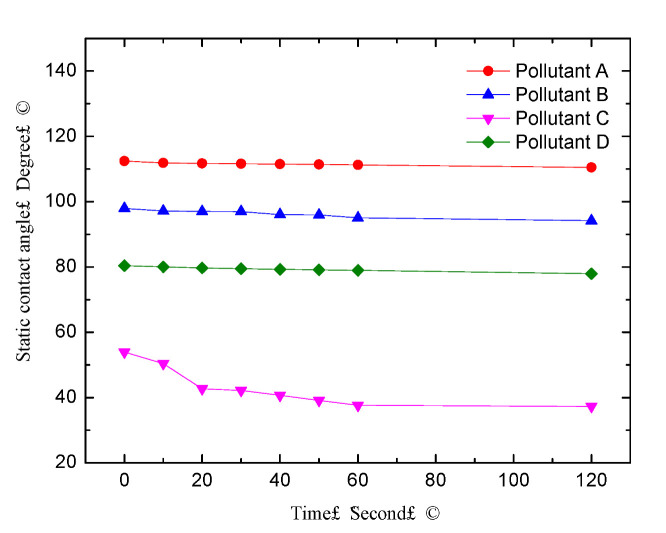
Contact angle lag phenomenon on the surface coated with different pollutants.

The contact angle lag phenomenon was a significant problem when the static contact angle method was used to characterize the hydrophobicity of polluted silicone rubber material. As the water drops to the solid surface, the contact angle gradually decreases over time due to gravity, water absorption, and other factors such as surface pollution. Any contact angle value between the receding contact angle and the advancing contact angle can keep the droplet relatively static and can be measured as a “static contact angle” under certain conditions. Hence, the “static contact angle” is not the only determining value [22]. Therefore, if the measurement time after dripping is different, it may cause a significant difference in the static contact angle measured on the same surface. Figure 3 shows the contact angle lag phenomenon on the surface of HTV silicone rubber coated with different pollutants. The surface of HTV silicone rubber was coated with different pollutants, and then the static contact angles were measured on different polluted surfaces. In Figure 3, each line represented the static contact angle measured on the different polluted surfaces varied with time. The static contact angle (the unit of angle was a degree, as shown in Figure 3) decreased considerably in the first 60 s after the drop and then decreased slowly with the time (the unit of the time was a second, as shown in Figure 3). Therefore, the static contact angle in this test was measured in the 60th second after the drop. This was measured with a repeatability of ±1.8° and was averaged over the (two) angles obtained from each of the ten droplets. The environment temperature was 30 °C, and the relative humidity was 45% during the experiments. The process of hydrophobicity transfer was observed over 144 h.

## 3. Experimental Results

### 3.1. Hydrophobicity Transfer of Inert Materials

The process of hydrophobicity transfer was determined in the presence of the already mentioned ten pollutants on the surface of HTV-SR samples (Figure 1). The deposits were the natural pollutants found on the surface of insulators near the sea and near a steel plant; powdered coal, charcoal, zinc oxide, aluminum, cement, and silica (to represent possible pollutants); kaolin and kieselguhr. Coal and charcoal dust could be present due to coal-burning factories and forest fires, metal oxides due to various industrial processes, cement due to being near cement factories, and silica due to dust storms.

**Figure 4:**
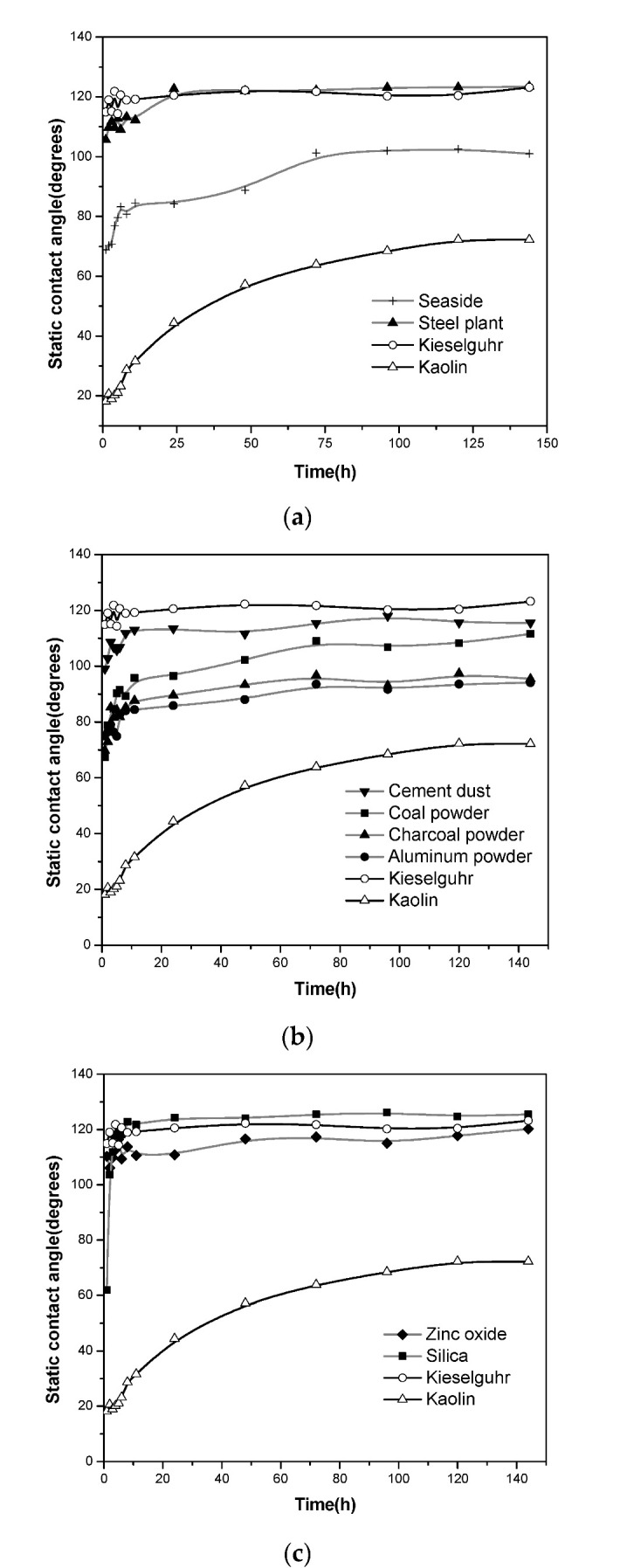
The transfer of hydrophobicity for ten types of inert pollutants: (**a**) from insulators within 1000 m of the sea and a steel plant; (**b**) coal, charcoal, aluminum powder, and cement dust; (**c**) zinc oxide and silica.

The results of contact angle variation with time are shown in Figure 4, where the contact angle was plotted against time. The static contact angle of the unpolluted HTV-SR sample was 105.2°, with an error band from −2.8% to +3.0%. In each graph, the curves for kieselguhr and kaolin are shown for comparison. In Figure 4a, the rates of hydrophobicity transfer are shown for HTV-SR samples coated with pollutants (as described in the previous section) gained by scraping from insulators near a steel plant and near the sea. Figure 4b shows the rate of hydrophobicity transfer when coal, cement dust, charcoal, and aluminum powder were used as pollutants. The rates of hydrophobicity transfer for two other inert materials, zinc oxide and silica, are shown in Figure 4c.

It is clear from Figure 4 that the ten different inert materials had different migration times and static contact angles. The migration characteristics of silica, natural pollution near a steel plant, and kieselguhr were the best, saturated within 20 h, and the final static contact angle could reach 120°. Among the ten inert materials, only zinc oxide, coastal natural pollution, and kaolin remained unsaturated after 40 h of migration. Kaolin had the worst migration characteristics, saturated only after 144 h of migration, and the final static contact angle was only 75°. The static contact angles of aluminum and charcoal were about 90°, better than kaolin, indicating a state between hydrophobicity and hydrophilicity. However, the final static contact angles of the two materials were about 20% higher than that of kaolin.

The hydrophobicity of the polluted silicone rubber insulator determined the degree of moisture resistance of the pollution layer. The droplet morphology on the surface of the dirty layer during moisture affected the distribution of the surface electric field and discharge characteristics [23,24]. The better the hydrophobic performance was, the more difficult the pollution layer was to be affected by moisture. The soluble salt in the pollution layer is not easily dissolved entirely, which inevitably leads to a rise in the pollution flashover voltage of the insulator. In reference [25], the negative DC pollution flashover voltage of composite insulators under hydrophobicity conditions was compared with that under hydrophilic conditions. The flashover gradient of the hydrophobicity specimen was nearly 2.53 times that of the hydrophilic specimen. It could be seen that the hydrophobic properties of composite insulators had significant effects on their pollution flashover voltage. Therefore, the hydrophobicity and insulation performance of the silicone rubber coated with a different pollution layer ensured the maximum pollution flashover voltage sustained in the electrical power system. The higher pollution flashover voltage could protect the insulator from pollution flashover in various environments and not lead to power failure.

The hydrophobic migration properties of the ten inert materials varied widely. Taking kieselguhr and kaolin, for example, the migration saturation time of kieselguhr was nearly 130 h faster than that of kaolin, and the final kieselguhr was about 60% higher than that of kaolin. To explain the influence of pollution material characteristics on the hydrophobic migration characteristics, the mechanisms of the hydrophobic migration characteristics were deeply studied and analyzed. Furthermore, the influence of pollution material characteristics on the hydrophobic migration characteristics was analyzed via SEM, specific surface area, and porosity.

### 3.2. Evidence of Small-Molecule Migration

In thermogravimetric analysis (TG), the mass of a sample is measured continuously during a heating process, and the mass changes occurring over a particular temperature range are associated with, e.g., thermal decomposition, degradation, and water loss [26].

**Figure 5:**
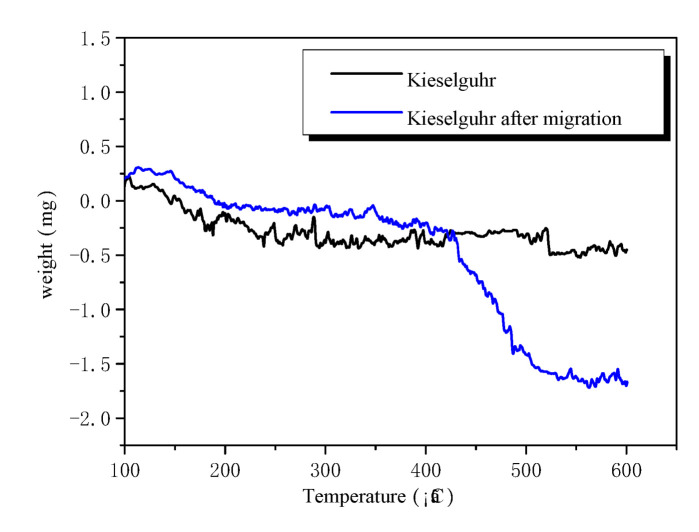
TG curve of kieselguhr before and after the hydrophobic migration test.

Kieselguhr was applied to the surface of HTV-SR. Kieselguhr before and after the migration was wiped down from the surface and used as samples for the thermogravimetric test. The migration time was more than 144 h, ensuring sufficient migration time for small molecules. Figure 5 shows the TG curve of kieselguhr before and after the hydrophobic migration test. It was revealed that the original samples of kieselguhr had no mass change range in TG analysis. In contrast, kieselguhr after the hydrophobic migration test had an obvious mass loss within the temperature range of 400–500 °C. The temperature range of the reaction gasification of the materials was similar to that of polydimethylsiloxane [27]. In other words, organic substances, likely polydimethylsiloxane, had migrated to kieselguhr from HTV silicone rubber over time.

Fourier transform infrared spectroscopy (FTIR) is often used to analyze the characteristic of the functional groups on the surface of materials. In the test performed in this study, the analysis instrument was a Spectrum GX Infrared Analyzer (PE). The principle of measurement was that the absorption frequency of infrared light by different functional groups in the analytical sample was different. When the frequency of continuously changing infrared light was irradiated to the analytical sample to be tested, an infrared spectrogram reflecting the characteristics of the functional groups contained in the analytical sample could be obtained.

**Figure 6:**
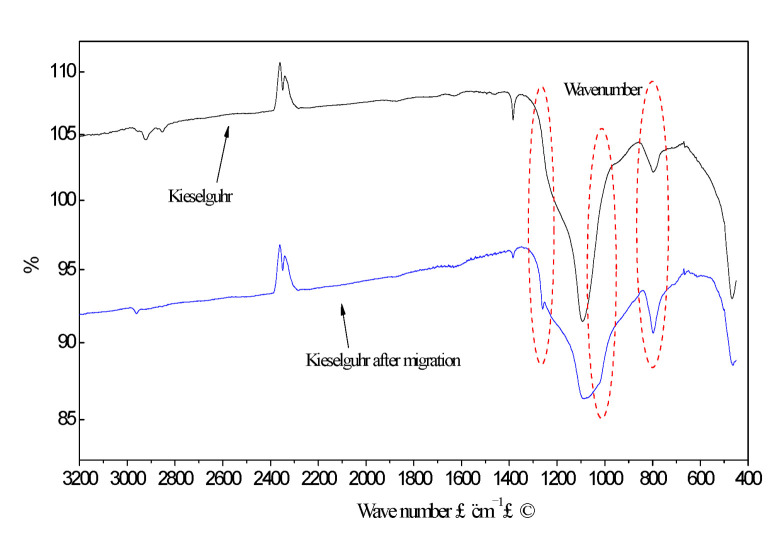
FTIR curve of kieselguhr before and after the hydrophobic migration test.

The kieselguhr before and after the migration was wiped down from the surface and used as samples for the FTIR test. The migration time was more than 144 h, which ensured sufficient migration time for small molecules. As shown in Figure 6, when the kieselguhr acquired hydrophobicity, the infrared spectrogram changed significantly from its initial state, manifesting in the wavenumber of 800 cm^−1^, 1100~1000 cm^−1^, and the more apparent infrared feature peak at 1250 cm^−1^. The infrared characteristic peak position of the functional groups represented the siloxane structure [28,29]. Therefore, the migration of siloxane in silicone rubber was an essential factor in the hydrophobic migration, which was consistent with TG results.

Gas chromatography–mass spectrometry (GC/GC–MS Turbomass/HP5973, Palo Alto, CA, USA) was also used to analyze the migration of small molecules to the polluted surface. Due to the sampling limitation of GC–MS analysis, hydrophobic particles could not be directly analyzed as the object of study, and it was necessary to use a carrier solution to extract organic substances from the polluted surface. The hydrophobic particles were soaked in the carrier solution, such as non-toxic n-hexane, and then the carrier solution was used for analysis. The results of mass spectrometry are listed in Table 1. The mass spectrometric analysis of the small molecules showed that these were the siloxane with the Si-O unit length of 8~12. When the above-mentioned small molecules migrated to the polluted surface from HTV silicone rubber, they made the polluted surface hydrophobic.

As detailed in the previous sections, thermogravimetric analysis (TG), Fourier transform infrared spectroscopy (FTIR), and gas chromatography–mass spectrometry (GC–MS) were used to analyze the migration of small molecules to the polluted surface. It was proven that small molecules in silicone rubber, such as siloxane, migrated to the surface of the pollution over time, and they could make the polluted surface hydrophobic, as shown in Section 3.1.

### 3.3. Influence of Pollution Material Characteristics

The evidence of small molecules migrating to the surface of the pollution over time was revealed in Section 3.2. The reason that ten different inert materials had different migration times and static contact angles might be due to the influence of pollution material characteristics on the characteristics of small-molecule migration. Therefore, the influence of pollution material characteristics on the characteristics of small-molecule migration was analyzed using SEM, specific surface area, and porosity, the results of which are detailed in the section.

**Figure 7:**
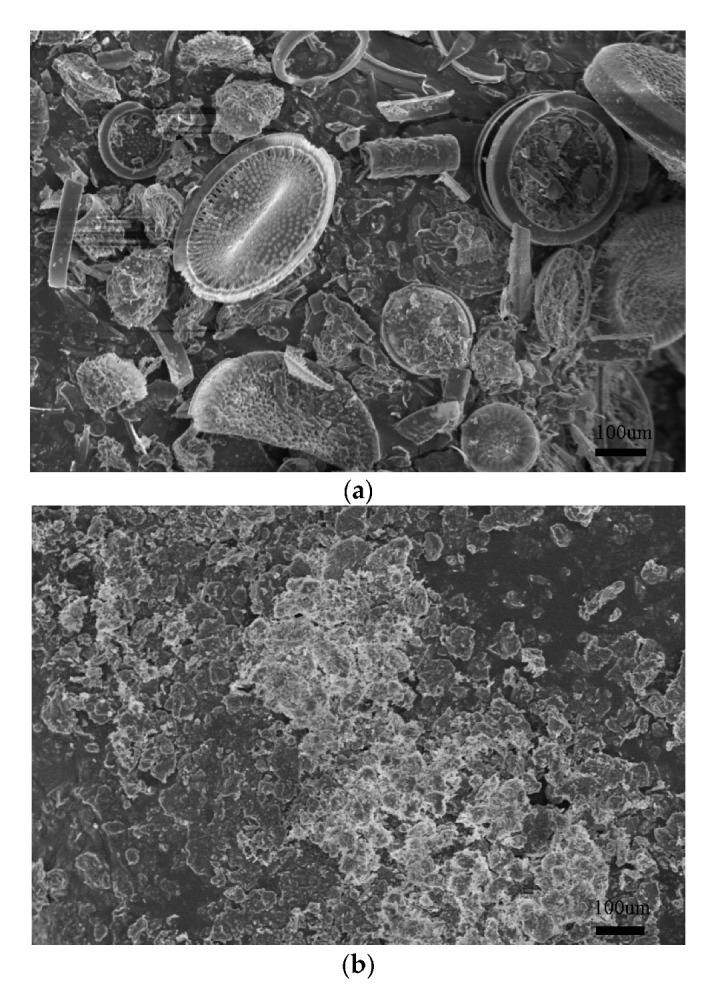
SEM photographs of pollutants: (**a**) kieselguhr; (**b**) kaolin.

High-resolution pictures of kieselguhr and kaolin were taken with a scanning electron microscope, as shown in Figure 7. It can be seen that kieselguhr is an aggregate of diatom skeletons with a wide variety of shapes and sizes. Due to their variety of shapes and sizes, they do not fit together and leave large voids between them. Hence, the LMW components of polysiloxane can spread and disperse through them relatively quickly and easily. By contrast, kaolin is seen to be of a more compact nature, and due to the molecular structure already described, tends to stack up in layers of flake-like microcrystals with fewer and smaller inter-crystal voids.

**Figure 8:**
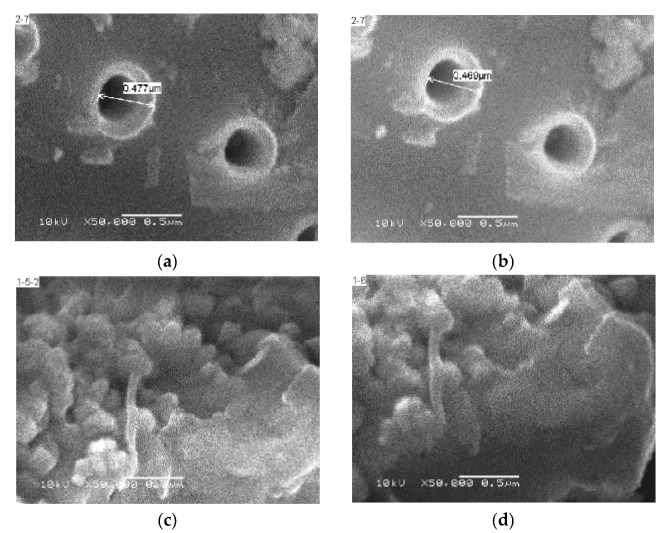
SEM photographs of pollutants before and after adsorption of small molecules: (**a**) kieselguhr before migration; (**b**) kieselguhr after migration; (**c**) kaolin before migration; (**d**) kaolin after migration.

SEM photographs of pollution before and after the adsorption of small molecules were also taken. The migration time was more than 144 h, ensuring sufficient migration time for small molecules. The SEM photographs of kieselguhr before migration and after migration are shown in Figure 8a,b, respectively. The pore size of the original kieselguhr was 0.477 μm, and that after migration of the small molecules was 0.469 μm. Hence, the pore size of kieselguhr decreased after the migration of small molecules. The SEM photographs of kaolin before migration and after migration are shown in Figure 8c,d, respectively. There was no noticeable difference in the physical morphology of kaolin before and after the migration of small molecules, but the morphology became smoother after the migration.

The transference of hydrophobicity is thus affected by the specific area and porosity of pollutants. The results of these measurements using an ASAP 2020 specific surface area and porosity analyzer are listed in Table 2. The specific area of kaolin was larger than that of kieselguhr. The pollution with a larger specific surface area needs to adsorb more small molecules to have similar hydrophobicity as that with a smaller specific surface area. This explains the experimental results in Figure 4 that the final degree of hydrophobicity for kaolin-polluted HTV-SR surfaces (contact angle 75°) was much less than those polluted with kieselguhr (contact angle 120°) under the same conditions (contact angle about 40% less). At the same time, the increase in the specific surface area of the pollution would increase the migration distance of the small molecules moving toward the contact surface of the pollution layer and air, which would also lead to a longer hydrophobic migration process. This also explains the experimental results in Figure 4 that the static contact angle was effectively at an asymptotic value for kaolin after 144 h. In contrast, kieselguhr had settled to an asymptotic value within 20 h.

## 4. Discussion

### 4.1. Solid Adsorption Theory

The results of SEM, specific area, and porosity revealed the influence of pollution material characteristics on the characteristics of small-molecule migration. The characteristics of small-molecule migration were strongly related to hydrophobic migration characteristics. Finally, the reasons why hydrophobic migration characteristics were affected by pollution material characteristics were explained. The differences in the hydrophobicity migration characteristics of inert materials in Section 3.1 were also explained in detail.

However, the reasons why small molecules migrate to the pollution material surface remained unclear. As revealed by the GC–MS analysis test, the hydrophobic substance on the polluted surface could fall off the polluted surface through ultrasonic vibration, so the adsorption of the small molecules by the polluted surface was only a physical adsorption process. Combined with the results of SEM, specific area, and porosity, the solid adsorption theory was adopted to explain why the small molecule would migrate to the pollution material surface.

The surface energy of silicone rubber was low, whereas the surface energy of the inert pollution material was high. From the perspective of energy balance and thermodynamics, when the pollution material came in contact with the silicone rubber material, there was a significant energy difference between the two interfaces. To weaken or eliminate the energy difference between the interface, a molecular movement process was initiated between the interface of the pollution material and silicone rubber material under the drive of thermal dynamics. Since the force between the molecules of the inert polluted substance was powerful, there would be no molecules moving away from the pollution surface to the silicone rubber surface. Therefore, the energy difference between the above interfaces would be eliminated by the migration of free small molecules of siloxane in silicone rubber to the pollution material. The results of the analysis and the experiment were in good agreement, indicating that our reasoning was correct.

Whether small siloxane molecules could detach from the silicone rubber body and move to the surface of pollution material depended on the capacity of pollution material to absorb small molecules. The adsorption capacity of solids is related to the material structure, such as specific surface area [30]. As revealed in Section 3.3, the characteristics of small-molecule migration were mainly affected by the material structure, which was measured using SEM, specific area, and porosity test. Hence, the conclusion derived from the solid adsorption theory was proved by the results of the test. Therefore, the solid adsorption theory well explained why small molecules would migrate to the surface of pollution material.

### 4.2. Equivalent Discussion of Natural and Artificial Pollution

From the analysis above, it is concluded that different pollution materials with different material structures have various adsorption capacities, leading to different migration times of the small molecule and static contact angles. The influence of pollution material characteristics on the characteristics of small-molecule migration was mainly the influence of material structure. If our analysis and solid adsorption theory were correct, mixtures of kaolin and kieselguhr would be able to simulate the values of almost all of the natural pollutants with different hydrophobicity transfer characteristics indicated in Section 3.1.

**Figure 9:**
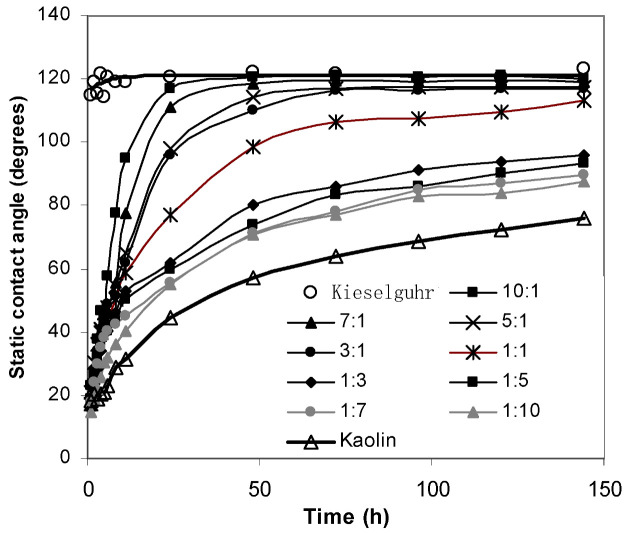
Transference of hydrophobicity for mixed pollutants.

Several mixtures of kieselguhr and kaolin were tested to determine the rate of hydrophobicity transfer. Mixtures were prepared with kieselguhr to kaolin ratios of 1:10, 1:7, 1:5, 1:3, 1:1, 3:1, 5:1, 7:1 and 10:1. The results with these mixtures are shown in Figure 9, together with the curves for pure kieselguhr and kaolin.

Clearly, the mixtures of the two inert materials—kaolin and kieselguhr—yielded a range of final (or saturation) values of the static contact angle, covering most of the naturally occurring pollutants (seaside and steel mill) as well as the simulated natural pollutants (coal, charcoal, and cement dust) and other possible contaminants tested (aluminum and zinc oxide powder), as shown in Figure 4. Only silica appeared to be slightly more hydrophobic than kieselguhr. Interestingly, the initially poor level of hydrophobicity (about 20°) remained approximately the same as that for 100% kaolin, even when the mixture consisted of 90% kieselguhr.

**Figure 10:**
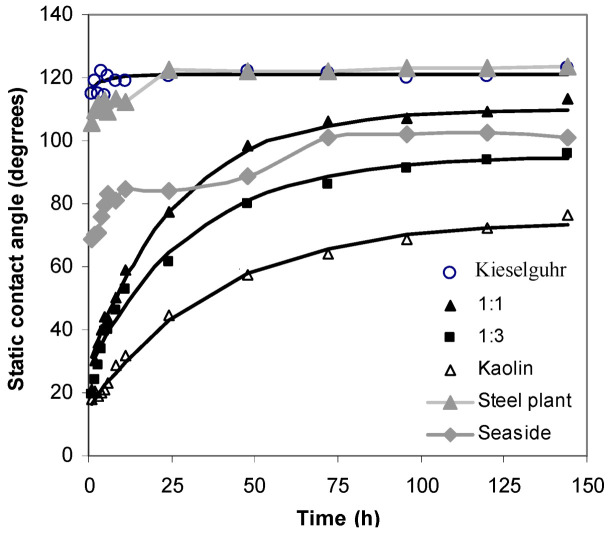
Equivalent investigation curve of natural and artificial pollutants.

It follows that, by appropriately choosing the mixture of the two standard test materials, kieselguhr and kaolin, the level of hydrophobicity of natural pollutants can be mimicked for testing composite insulators under polluted conditions. However, the insulators under test would need to be left for one to two days before testing to allow the completion of the hydrophobicity migration, since the characteristic times for the contaminants originating from the sea and steel mills are different. To illustrate this, the curves for seaside and steel mill pollution are replotted in Figure 10, together with the curves for mixtures of 1:1, 3:1 kaolin to kieselguhr (50% and 75% kaolin, respectively), and 100% kieselguhr. The tests were performed with the mixed soil of kaolin and kieselguhr, and two kinds of mill pollutions were measured under the same condition.

The stable contact angle for natural pollutants near a steel plant was about 120°, about 20% greater than that of the seaside pollutants due to the different nature of the deposits. Nevertheless, the former had an asymptotic or stable contact angle close to kieselguhr, and the latter had a contact angle falling within the range of the mixtures’ stable contact angles. This allows pure kieselguhr to simulate the pollutants near a steel plant and a mixture of about 60% kaolin and 40% kieselguhr to simulate the pollution deposits near the sea.

The time for hydrophobicity transference to a surface contaminated by natural pollutants from the vicinity of a steel plant only needed about 25 h to reach saturation. However, with seaside pollutants, the transfer process required 75 h. On the other hand, the pollution mixtures exhibited transfer times within about 20 to 80 h to reach the stable static contact angle (within 10%). Thus, before testing a mixture of kieselguhr and kaolin intended to represent a particular type of natural pollutant, the polluted insulators would need to “rest” for 1 to 4 days to ensure that they had reached their asymptotic or stable contact angle.

In conclusion, the mixtures of kaolin and kieselguhr could simulate almost all of the natural pollutants with different hydrophobicity transfer characteristics. Hence, it was proven that the hydrophobic migration characteristics were mainly affected by pollution material structure. It was also confirmed again that the solid adsorption theory could be adopted to explain why small molecules would migrate to the pollution material surface. Furthermore, it was also revealed that the hydrophobicity migration characteristics of mixtures of kaolin and kieselguhr enable them to be used as artificial pollutants instead of natural pollutants in the pollution flashover voltage test.

## 5. Conclusions

(1) The ten different inert materials analyzed in this study had different migration times and static contact angles. The hydrophobicity transfer process for kaolin-polluted HTV-SR surfaces was much slower than those polluted with kieselguhr under the same conditions (about 7 times slower). The final degree of hydrophobicity for kaolin-polluted HTV-SR surfaces (contact angle 75°) was much less than that for kieselguhr-polluted surfaces (contact angle 120°) under the same conditions (contact angle about 40% less).
(2) Thermogravimetric analysis (TG), Fourier transform infrared spectroscopy (FTIR), and gas chromatography–mass spectrometry (GC–MS) were used to analyze the migration of small molecules to the polluted surface. The evidence of small molecules migrating to the surface of the pollution over time was found.
(3) The microstructure of the pollutants influenced the hydrophobicity transfer characteristics of HTV-SR. Kieselguhr comprises a wide variety of siliceous diatom skeletons of different shapes and sizes. This loose structure inevitably has large voids between the diatom skeletons, which allow the swift migration of LMW components of polysiloxane through the kieselguhr material. In contrast, the similar flat-sheet-like crystals of kaolin stack together with fewer interstitial voids, resulting in slower hydrophobicity transfer.
(4) Mixtures of kieselguhr and kaolin can be used to emulate natural pollutants in testing composite insulators. A rest period of one to four days is required before the test to complete the hydrophobicity transfer.

## Figures and Tables

**Table 1:** Value *n* of the small molecules obtained via GC–MS mass spectrometry.

Chromatographic Separation Time	8.96	10.66	12.2	13.63	14.91	16.11	…	23.55	25.24
Value *n*	8	9	10	11	12	13	…	20	21

**Table 2:** Specific area and porosity of kaolin and kieselguhr.

Material	Kaolin	Kieselguhr
Specific area (m^2^/g)	19	14
Total pore volume (mm^3^/g)	54	37
Average pore (nm)	11.2	10.9

## Data Availability

Not applicable.

## References

[1] Salem A.A., Lau K.Y., Rahiman W., Al-Gailani S.A., Abdul-Malek Z., Rahman R.A., Al-Ameri S.M., Sheikh U.U. (2021). Pollution Flashover Characteristics of Coated Insulators under Different Profiles of Coating Damage. Coatings.

[2] Ghayedi M., Shariatinasab R., Mirzaie M. (2021). AC flashover dynamic theoretical and experimental model under fan-shaped and longitudinal pollution on silicone rubber insulator. IET Sci. Meas. Technol..

[3] Chrzan K.L., Brzezinski H.M. (2021). Anomalous flashovers of silicone rubber insulators under the artificial rain test. Arch. Electr. Eng..

[4] He S., Wang J., Hu J., Zhou H., Nguyen H., Luo C., Lin J. (2019). Silicone rubber composites incorporating graphitic carbon nitride and modified by vinyl tri-methoxysilane. Polym. Test..

[5] Liao Y., Weng Y., Wang J., Zhou H., Lin J., He S. (2019). Silicone Rubber Composites with High Breakdown Strength and Low Dielectric Loss Based on Polydopamine Coated Mica. Polymers.

[6] Jin L., Ai J., Han S., Zhou G. (2021). Probability Calculation of Pollution Flashover on Insulators and Analysis of Environmental Factors. IEEE Trans. Power Deliv..

[7] Chang J.W., Gorur R.S. (1994). Surface recovery of silicone rubber used for HV outdoor insulation. IEEE Trans. Dielectr. Electr. Insul..

[8] Li Z., Yin F., Cao B., Wang L., Shao S., Farzaneh M. (2020). Pollution flashover performance of RTV coatings with partial damage. Int. J. Electr. Power Energy Syst..

[9] Tahir M.H., Arshad A., Manzoor H.U. (2022). Influence of corona discharge on the hydrophobic behaviour of nano/micro filler based silicone rubber insulators. Mater. Res. Express.

[10] Khattab T.A., Mowafi S., El-Sayed H. (2019). Development of mechanically durable hydrophobic lanolin/silicone rubber coating on viscose fibers. Cellulose.

[11] Mendoza A.I., Moriana R., Hillborg H., Strömberg E. (2019). Super-hydrophobic zinc oxide/silicone rubber nanocomposite surfaces. Surf. Interfaces.

[12] Liu H., Cash G.A., Sovar R.D., George G.A., Birtwhistle D. (2006). Studies of the diffusion of low molecular weight silicone fluids on polluted hv silicone insulators. I. use of diffuse reflectance FTIR. IEEE Trans. Dielectr. Electr. Insul..

[13] Sakoda T., Tabira K., Miyake T., Hayashi N., Haji K., Aka Y., Fukano T. (2018). Discharge behavior and dielectric performance of artificially polluted hydrophobic silicone rubber. J. Electrost..

[14] Sarkarat M., Lanagan M., Ghosh D., Lottes A., Budd K., Rajagopalan R. (2020). High field dielectric properties of clay filled silicone rubber composites. Mater. Today.

[15] Myat Thu A., Song M., Wu S., Sheng A., Chen X., Wang X. (2019). Artificial Neural Network Prediction and Mechanism Analysis for Migration of Environmental Contaminant Cyclic Organosiloxane Oligomer from Silicone Rubber. Ind. Eng. Chem. Res..

[16] (1991). Artificial Pollution Tests on High-Voltage Insulators to Be Used on a.c. Systems.

[17] Yong Z. (2019). Influence of corona discharge on hydrophobicity of silicone rubber used for outdoor insulation. Polym. Test..

[18] Kumar V., Lee D.J. (2017). Effects of thinner on RTV silicone rubber nanocomposites reinforced with GR and CNTs. Polym. Adv. Technol..

[19] Swift D.A., Spellman C., Haddad A. (2006). Hydrophobicity Transfer from Silicone Rubber to Adhering Pollutants and its Effect on Insulator Performance. IEEE Trans. Dielectr. Electr. Insul..

[20] (2005). Polymeric Insulators for Indoor and Outdoor Use with Nominal Voltage > 1000 V.

[21] (2003). Guidance on the Measurement of Wettability of Insulator Surfaces.

[22] Kumar V., Alam M.N., Manikkavel A., Song M., Lee D.J., Park S.S. (2021). Silicone rubber composites reinforced by carbon nanofillers and their hybrids for various applications: A review. Polymers.

[23] Cheng Z.X., Liang X.D., Zhou Y.X., Wang S.W., Guan Z.C. Observation of corona and flashover on the surface of composite insulators. Proceedings of the IEEE Power Tech Conference.

[24] Lan L., Gorur R.S. (2008). Computation of ac wet flashover voltage of ceramic and composite insulators. IEEE Trans. Dielectr. Electr. Insul..

[25] Yongqing Y.U. (2006). DC Contamination Flashover Performance of Composite Insulators Lost Hydrophobicity. Power Syst. Technol..

[26] Zhu L.Y., Sun Y.L., Chen G.Y. (1997). Handbook of Instrument Analysis.

[27] Camino G., Lomakin S.M., Lazzari M. (2001). Polydimethylsiloxane thermal degradation Part 1. Kinetic aspects. Polymer.

[28] Kim S.H., Chereny E.A., Hackam R., Rutheford K.G. (1994). Chemical changes at the surface of RTV silicone rubber coatings on insulators during dry-band arcing. IEEE Trans. Dielectr. Electr. Insul..

[29] Kim S.H., Chemey E.A., Hackam R. (1990). The loss and recovery of hydrophobicity of RTV silicone rubber insulator coatings. IEEE Trans. Power Deliv..

[30] Yan J., Zhang Q., Gao J. (1986). Adsorption and Coagulation.

